# Effect of *Apis mellifera syriaca* Bee Venom on Glioblastoma Cancer: In Vitro and In Vivo Studies

**DOI:** 10.3390/molecules29163950

**Published:** 2024-08-21

**Authors:** Charbel Chahla, Mohamad Rima, Charbel Mouawad, Rabih Roufayel, Hervé Kovacic, Dany El Obeid, Jean-Marc Sabatier, José Luis, Ziad Fajloun, Bilal El-Waly

**Affiliations:** 1 Inst Neurophysiopathol (INP), CNRS, Aix-Marseille Université, 13385 Marseille, France; charbelchahla1997@gmail.com (C.C.); herve.kovacic@univ-amu.fr (H.K.); 2 Department of Natural Sciences, Lebanese American University, Byblos P.O. Box 36, Lebanon; mohamad.rima@lau.edu.lb; 3 Laboratoire d’Histologie Embryologie Biologie de la Reproduction CECOS, Assistance Publique-Hôpitaux Universitaires Paris Centre, CHU Cochin, 75014 Paris, France; charbelmawad11@outlook.com; 4 College of Engineering and Technology, American University of the Middle East, Egaila 54200, Kuwait; rabih.roufayel@aum.edu.kw; 5 Faculty of Agriculture & Veterinary Sciences, Lebanese University, Dekwaneh, Beirut 1100, Lebanon; dany.elobeid@ul.edu.lb; 6 Laboratory of Applied Biotechnology (LBA3B), Azm Center for Research in Biotechnology and Its Applications, Department of Cell Culture, EDST, Lebanese University, Tripoli 1300, Lebanon; ziad.fajloun@ul.edu.lb; 7 Department of Biology, Faculty of Sciences 3, Campus Michel Slayman Ras Maska, Lebanese University, Tripoli 1352, Lebanon

**Keywords:** glioblastoma, *Apis mellifera*, *Apis mellifera syriaca*, animal venom, cytotoxicity, in vitro, in vivo, anti-cancer, therapeutic efficacy, cell viability

## Abstract

Glioblastoma multiforme (GBM) is a highly aggressive and fatal primary brain tumor. The resistance of GBM to conventional treatments is attributed to factors such as the blood–brain barrier, tumor heterogeneity, and treatment-resistant stem cells. Current therapeutic efforts show limited survival benefits, emphasizing the urgent need for novel treatments. In this context, natural anti-cancer extracts and especially animal venoms have garnered attention for their potential therapeutic benefits. Bee venom in general and that of the Middle Eastern bee, *Apis mellifera syriaca* in particular, has been shown to have cytotoxic effects on various cancer cell types, but not glioblastoma. Therefore, this study aimed to explore the potential of *A. mellifera syriaca* venom as a selective anti-cancer agent for glioblastoma through in vitro and in vivo studies. Our results revealed a strong cytotoxic effect of *A. mellifera syriaca* venom on U87 glioblastoma cells, with an IC50 of 14.32 µg/mL using the MTT test and an IC50 of 7.49 µg/mL using the LDH test. Cells treated with the bee venom became permeable to propidium iodide without showing any signs of early apoptosis, suggesting compromised membrane integrity but not early apoptosis. In these cells, poly (ADP-ribose) polymerase (PARP) underwent proteolytic cleavage similar to that seen in necrosis. Subsequent in vivo investigations demonstrated a significant reduction in the number of U87 cells in mice following bee venom injection, accompanied by a significant increase in cells expressing caspase-3, suggesting the occurrence of cellular apoptosis. These findings highlight the potential of *A. mellifera syriaca* venom as a therapeutically useful tool in the search for new drug candidates against glioblastoma and give insights into the molecular mechanism through which the venom acts on cancer cells.

## 1. Introduction

“Gliomas” is an umbrella term used to designate all forms of central nervous system tumors arising from glial cells. Gliomas are categorized into four grades, with each successive grade associated with increased aggressiveness and mortality rates. These tumors are heterogeneous in nature, originating from various types of glial cells [1,2]. Glioblastoma or glioblastoma multiforme (GBM) is a grade VI astrocytoma (astrocyte glioma), and is the most aggressive and malignant form of adult primary brain cancer [3,4]. GBM is linked with poor outcomes in patients and an average survival of just one year following diagnosis [5,6]. Glioblastoma has a fast-progression curve with a median survival duration of about 16 months [7]. It is the most common primary brain cancer in adults and typically arises de novo without any previous evidence of lower-grade gliomas [8]. The conventional treatment for GBM involves a combination of radiotherapy, surgery, and temozolomide chemotherapy [9]. However, most patients face tumor progression, leading to inevitable mortality. This multimodality regimen used for glioblastomas is consistently associated with a poor prognosis, mainly due to the inherent resistance of the disease [10]. Thus, new therapeutic approaches must be devised to effectively tackle this issue.

Addressing the challenges in developing new therapeutic tools for GBM is a complex task given the numerous obstacles that present significant difficulties in the process: First, the efficacy of chemotherapy for glioblastoma is limited by the blood–brain barrier, which prevents drugs from reaching the tumor site and targeting the affected glial cells [11]. Second, numerous studies have highlighted the heterogeneity of glioblastoma, both between patients and at the intratumoral level, as a major obstacle to achieving satisfactory therapeutic results [12]. The diverse cellular composition and genetic mutations within glioblastoma tumors contribute to treatment resistance and the failure of multiple therapeutic approaches [13]. Third, the presence of glioblastoma stem cells further complicates treatment efficacy, as these cells are known to contribute to tumor recurrence and resistance to therapy [14].

Various drug combinations and novel treatment approaches have been explored to overcome these limitations. For instance, the combination of temozolomide and thalidomide has shown improved efficacy in glioblastoma patients compared to single therapies [15,16]. Furthermore, the use of tumor-treating fields (TTFs) has emerged as an alternative treatment modality, showing comparable efficacy to standard chemotherapeutic agents in the treatment of recurrent glioblastoma [17].

Despite these efforts, the development of effective therapeutics for glioblastoma remains a critical unmet need. The lack of effective therapies is evident in the limited survival benefits observed in patients, emphasizing the urgency for the development of novel treatment strategies [18]. The complexity of glioblastoma pathogenesis and resistance mechanisms requires a multidisciplinary approach to meet the challenges associated with treatment limitations [19].

The use of natural anti-cancer extracts has garnered significant attention due to their potential therapeutic benefits. Natural products, including plant extracts and compounds, have been explored for their anti-cancer properties, offering promising avenues for the development of novel anti-cancer drugs [20,21]. These natural extracts have shown potential in modulating cell proliferation, inducing apoptosis, and regulating gene expression in cancer cells, thereby presenting as attractive candidates for anti-cancer drug development [22,23,24,25]. Furthermore, the combination of natural extracts with conventional chemotherapeutic regimens has been proposed to enhance the safety and efficacy of cancer treatment [26].

Animal venoms have emerged as important sources of anti-cancer agents [27,28,29]. Indeed, these animal extracts, including bee venom, have also been investigated for their therapeutic potential in the cancer treatment field [30]. The processing and refinement of animal venoms have led to the development of pharmacopuncture injections, which have shown promise in medical usage, including cancer therapy [31]. Numerous studies have reported the cytotoxic effects of bee venom on cancer cells, suggesting its potential as a selective anti-cancer agent [30,32].

Although research into the effects of natural extracts on glioblastoma is ongoing, studies have already indicated the potential of certain plant extracts, such as Ginkgo biloba leaf extract, in regulating cell proliferation and inducing cell death in glioblastoma cells [33]. In addition, the development of polymersomes has facilitated combination therapy for glioblastoma, highlighting the potential of complex drug delivery systems in improving the treatment of this challenging cancer [34]. Additionally, the potential of venom-based drugs, including bee venoms, in targeting tumor-intrinsic vulnerabilities of glioblastoma is showing promising results, either alone [35] or in combination with conventional anti-cancer drugs [36,37]. Together these findings highlight the diverse avenues currently being explored for the treatment of this aggressive cancer through discovering a new set of drugs or by boosting the efficiency of current drugs. Other studies have reported the anti-cancer properties of melittin, the principal active component of bee venom, and highlighted an inhibitory effect of the venom on matrix metalloproteinase secretion, which is known to promote cancer invasion [36].

The venom of the Middle Eastern bee, *Apis mellifera syriaca*, has been the subject of several of our previous studies where we investigated its proteomic content and biological properties. This venom has some significant biological properties with pharmaceutical relevance, including anticoagulant [38], antibacterial [39], and anti-cancer activities [38,40,41]. Our proteomic analysis confirmed the presence of many bioactive molecules including apamin, melittin, phospholipase A2 (PLA2), mast cell degranulating peptide (MCD-peptide), and hyaluronidase (Table 1). The two major constituents of the venom are melittin and PLA2, and they have been reported to be responsible for the majority of the bioactivities cited above [40]. The anti-cancer effect was mainly validated on various types of cancer including breast and colon cancer, but no study investigated the venom's effect on glioblastoma [38,40,41]. In addition, these studies were limited to in vitro models and require further validation in vivo since this cancer’s fatality relies on its microenvironment. Therefore, in this study, we aimed to further explore the valuable anti-cancer potential of *A. mellifera syriaca* venom on one of the most fatal types of cancer: glioblastoma. Starting with an in vitro screening of its anti-cancer activity on glioblastoma cells, we then developed a mouse model mimicking the tumor in vivo and dissected the molecular pathway through which the venom induces its toxicity. Our study explored *A. mellifera syriaca* venom's therapeutic efficacy and ability to address the complex challenges linked to glioblastoma treatment.

## 2. Results

### 2.1. In Vitro Effect of A. mellifera syriaca Venom on Glioblastoma Cancer Cell Lines

**Figure 1:**
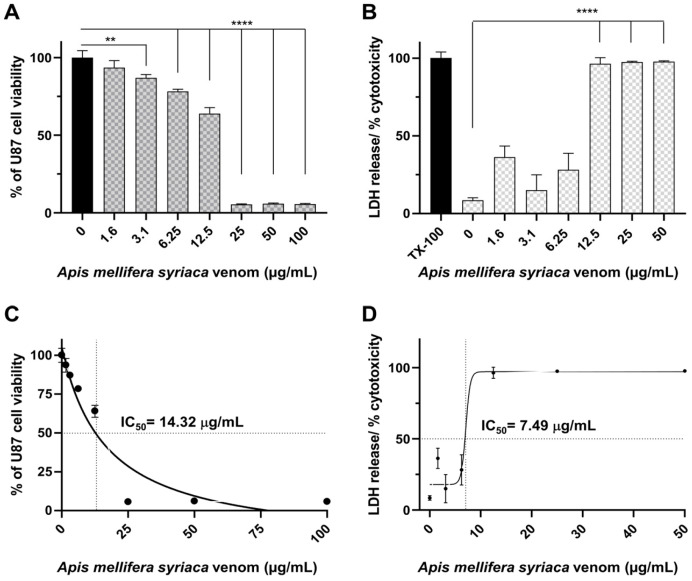
Cytotoxicity of *A. mellifera syriaca* venom on U87 glioblastoma cells in vitro. Cell viability of U87 glioblastoma cells was measured using (**A**) MTT and (**B**) LDH assays after treatment with increasing concentrations of *A. mellifera syriaca* venom. Results are expressed as mean ± SD of three independent experiments (*n* = 3). One-way ANOVA-test: **** *p* < 0.0001, ** *p* < 0.01. Curves for (**C**) MTT and (**D**) LDH assays showing IC50 = 14.32 μg/mL and 7.49 μg/mL, respectively.

The cytotoxic activity of *A. mellifera syriaca* venom on human glioblastoma U87 cells was studied using MTT (3-(4,5-dimethylthiazol-2-yl)-2,5-diphenyl tetrazolium bromide) and LDH (lactate dehydrogenase) assays. U87 cells were exposed to increasing concentrations of *A. mellifera syriaca* venom (1.56, 3.125, 6.25, 12.5, 25, 50, and 100 µg/mL). For the MTT assay, the results are expressed as a percentage of cell viability relative to untreated control cells with 100% viability, whereas for the LDH assay, the results are expressed as a percentage of cell toxicity compared to control cells treated with both Triton X-100 (TX-100) and untreated with the venom. For the MTT assay, the data obtained revealed that the *A. mellifera syriaca* venom almost completely inhibited the cell viability of U87 glioblastoma cells at concentrations equal to and above 25 µg/mL. This concentration induced almost 95% mortality of the treated cells compared with the untreated control (Figure 1A). These results were further supported by the LDH assay, which showed that the venom was able to induce significant cytotoxic effects on U87 cells starting from a concentration of 12.5 µg/mL (Figure 1B). These results obtained from the two cytotoxicity tests, the MTT and LDH assays, therefore reveal the strong cytotoxic effect of venom from the Middle Eastern bee *A. mellifera syriaca* on U87 cells, with IC50 values of 14.32 µg/mL (Figure 1C) and 7.49 µg/mL (Figure 1D), respectively.

**Figure 2:**
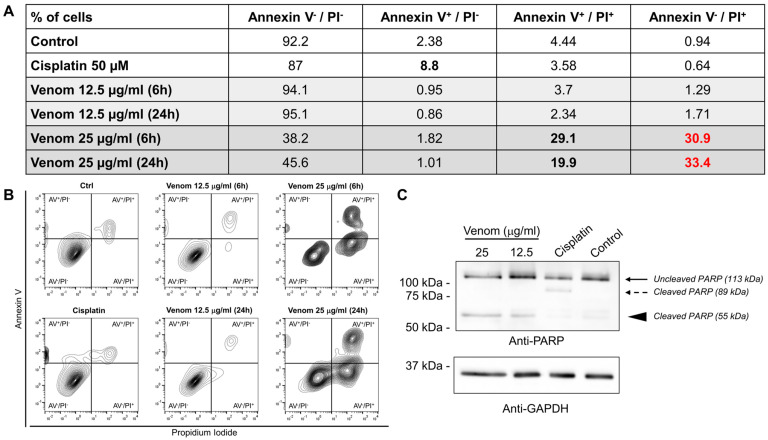
Molecular pattern of cell death induced by *A. mellifera syriaca* venom. (**A**) Summary table and (**B**) representative plots of annexin V (AV)/propidium iodide (PI) staining of U87 cells treated with different concentrations of *A. mellifera syriaca* venom and assessed for apoptosis induction. Cisplatin, which is known to induce apoptosis, was used as the positive control. (**C**) Western blot analysis of U87 cells treated with different concentrations of *A. mellifera syriaca* venom and assessed for PARP cleavage, a marker of apoptosis. Cisplatin, which is known to induce apoptosis, was used as the positive control. GAPDH was used as the loading control.

Next, we wanted to dissect the mechanism through which the venom acts on these cells. To this end, the apoptosis and necrosis profiles of U87 cells were evaluated using annexin V/propidium iodide (PI) following incubation with the venom. The two concentrations (12.5 and 25 µg/mL) that showed high toxicity in the MTT and LDH tests were used at two different time points (6 and 24 h) post-treatment. At 12.5 µg/mL, the venom did not show any apoptotic or necrotic effect on U87 cells, as indicated by the results being comparable to those of the control condition (Figure 2A,B). However, at 25 µg/mL, significant increases in annexin V^+^/PI^+^ cells and annexin V^−^/PI^+^ cells were observed (Figure 2A,B). The fact that a high proportion of the cells (~30%) were able to incorporate the PI without any annexin V^+^ staining suggests that the membrane was becoming permeable to the PI dye. These results suggest that upon venom treatment, the cellular membrane was disrupted in a similar fashion to that observed during necrosis. The increase in annexin V^+^/PI^+^ cells supports this idea, as annexin V^+^/PI^+^ cells are late apoptotic and necrotic cells. In addition, these results were not comparable to those of the cisplatin-treated cells, which showed an increase in annexin V^+^/PI^−^ cells, which are early apoptotic cells (Figure 2A,B). These results suggest that *A. mellifera syriaca* venom induces cytotoxic effects on U87 cells by disrupting their plasma membranes, leading to necrosis and/or apoptosis.

To investigate this hypothesis and to confirm the absence of conventional apoptotic pathway activation upon venom treatment, we assessed the cleavage of PARP, which is known to be cleaved into two fragments (89 kDa and 24 kDa) during apoptosis [42]. The Western blot results showed the apoptotic cleavage of PARP when the cells were treated with cisplatin, which is known to induce apoptosis (Figure 2C, dashed arrow). However, cells treated with the *A. mellifera syriaca* venom showed a cleaved PARP peptide with a size of 55 kDa (Figure 2C, arrowhead). This major 55 kDa fragment is normally seen when PARP is processed during necrosis [42]. Taken together, these findings show that *A. mellifera syriaca* venom induces necrosis of U87 cells in vitro.

### 2.2. In Vivo Effect of A. mellifera syriaca Venom in Human Glioblastoma Mouse Model

**Figure 3:**
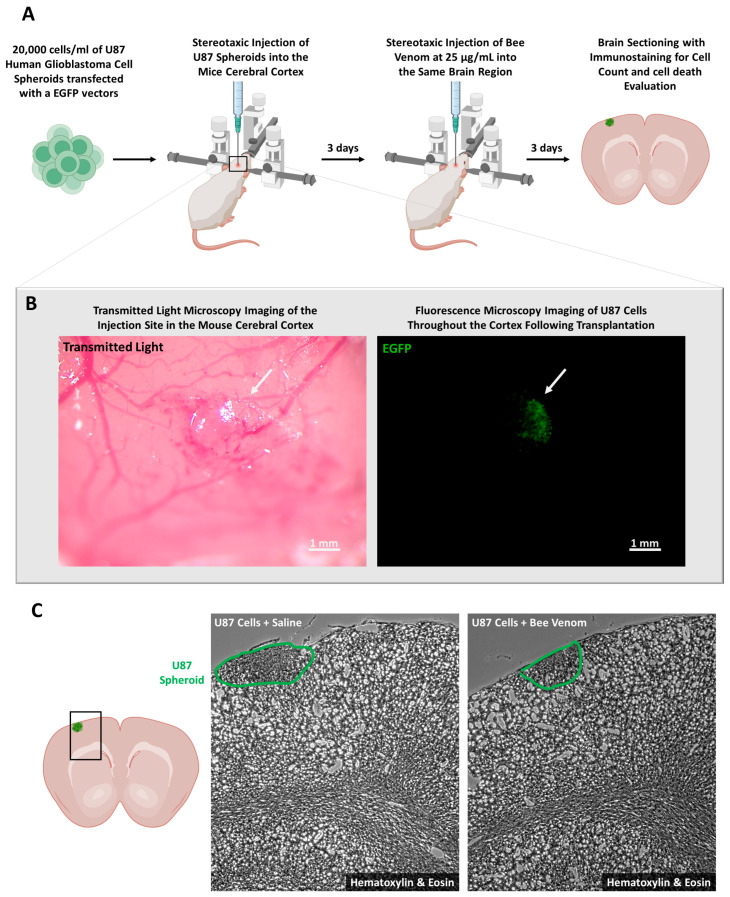
In vivo experimental workflow and preliminary assessment of bee venom cytotoxicity. (**A**) Schematic representation of the experimental timeline for our in vivo study. U87 cells were cultured and transfected in vitro with a plasmid to express EGFP under the control of a CMV promoter. Subsequently, 20,000 of these cells were stereotaxically injected into the cerebral cortex. After a 3-day incubation period, during which the cells proliferated, the mice received either saline or 25 µg/mL of *A. mellifera syriaca* venom. Three days post-treatment, the mice were sacrificed, and brain tissues were collected for further analysis, including immunohistochemical staining to detect EGFP. (**B**) Cellular Localization: Utilizing transmitted light, this panel displays the cells injected into the cerebral cortex. These cells fluoresce in the 488 channel, which was used to determine their specific location and presence during the injection process. (**C**) Hematoxylin and Eosin Staining: (**Left**) A schematic representation of the regions where hematoxylin and eosin staining was performed. (**Right**) Hematoxylin and eosin staining images comparing U87 cells treated with saline or bee venom 3 days post-treatment. The staining shows that the size of the cellular mass was significantly smaller after treatment with bee venom.

For our in vivo study, we employed the experimental procedure illustrated in Figure 3A. The initial steps involved culturing U87 cells in vitro, followed by meticulous transfection with a plasmid harboring the enhanced green fluorescent protein (EGFP) gene under the precise control of a cytomegalovirus (CMV) promoter. Subsequently, 20,000 EFGP-transfected cells were injected into the cerebral cortex of mice using a stereotaxic approach. A critical 3-day incubation period allowed for the integration of the cells into the surrounding tissues and the initiation of proliferative activities. The transfection efficiency and localization of EGFP-expressing cells within the brain tissue were verified using immunohistochemical techniques (Figure 3B). Transmitted light images revealed the presence of injected cells, while the 488-channel fluorescence showed the specific localization of EGFP-expressing cells during the injection process (Figure 3B). Following the 3-day incubation period, the mice were subjected to treatment with either saline or *A. mellifera syriaca* venom at a concentration of 25 µg/mL, as optimized through our prior in vitro investigations. After an additional 3-day period, the experimental subjects were humanely sacrificed, and brain tissue collection was executed after fixation. Histological analysis using hematoxylin and eosin staining was then employed to measure the tumor size after bee venom injection. The microscopic observations showed that the size of the cellular mass was significantly smaller in bee venom-treated mice compared to control saline-treated mice (Figure 3C). These findings suggest that *A. mellifera syriaca* venom reduces glioblastoma tumor size in vivo.

**Figure 4:**
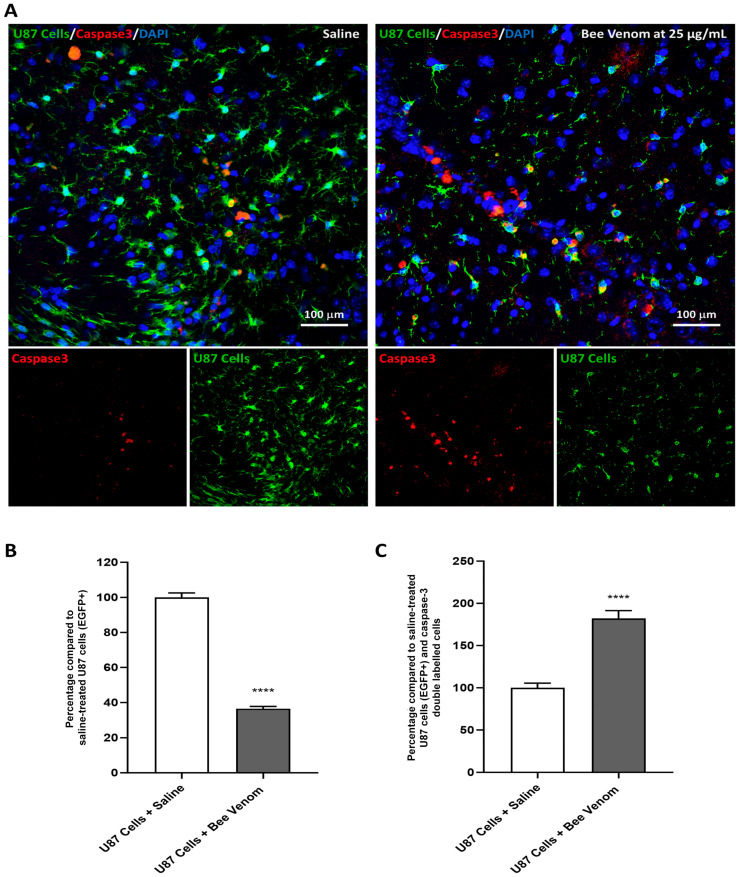
Bee venom cytotoxicity in vivo and its corresponding molecular mechanism. (**A**) Representative immunofluorescence results showing U87 cells (EGFP^+^, green), caspase-3 expressing cells (red), and nuclei (blue). The left panel represents the saline-injected condition, while the right panel illustrates the A. mellifera syriaca bee venom (25 µg/mL)-injected condition 3 days post-injection. (**B**) Quantification of EGFP^+^ U87 cells showing a significant decrease in the number of EGFP^+^ cells 3 days after bee venom injection compared to the saline-injected condition. (**C**) Quantification of double-positive (EGFP^+^/caspase-3^+^) cells as a percentage, demonstrating a notable increase in the bee venom-injected group, which is indicative of the occurrence of apoptosis. The statistical analysis was conducted using the Student’s *t*-test, and all data are presented as mean ± standard error of the mean (SEM). The significance level was set at *p* < 0.0001 (****). The results represent a comprehensive assessment of *n* = 5 mice per experimental condition.

To further validate the in vivo cytotoxic potential of the venom and to investigate its corresponding mechanism of action, we combined EGFP and caspase-3 immunolabeling of brain tissues. This analysis enabled the assessment of the number of EGFP-expressing cells and the potential signaling pathway involved in responding to the administered treatments. Green fluorescence indicated EGFP-expressing U87 cells and served as a vital marker for the transplanted cell population, while red fluorescence marked cells expressing caspase-3, a well-established indicator of cellular apoptosis. Three 20X fields within the injected cell sphere for each section were analyzed, and a total of three sections per mouse were meticulously considered, encompassing data from five mice per experimental condition. The left panel in Figure 4A represents the condition where saline was administered 3 days post-transplantation, while the right panel illustrates the response following the injection of *A. mellifera syriaca* bee venom.

The immunostaining analysis revealed a pronounced and statistically significant reduction in the number of U87 cells expressing EGFP (EGFP^+^) 3 days post-bee venom injection when compared to the saline-injected group (Figure 4B). This notable decrease aligned with a parallel and significant surge in the number of cells expressing caspase-3 (Figure 4C). Furthermore, an increase in the number of cells positive for both caspase-3 and EGFP was observed, indicating a clear association between bee venom administration and suggest cellular apoptosis within the U87 sphere. These data validate the cytotoxic potential of *A. mellifera syriaca* bee venom on an in vivo glioblastoma mouse model and lends additional depth to our understanding of the interplay between bee venom and the U87 cell population, particularly in the context of apoptosis modulation. Collectively, these results underscore the pivotal role of *A. mellifera syriaca* bee venom in influencing cellular dynamics and apoptosis within the U87 glioblastoma model.

## 3. Discussion

*A. mellifera syriaca* bee venom is able to exert different biological properties that can be beneficial for pharmaceutical research. In this study, we explored the anti-cancer potential of *A. mellifera syriaca* venom on one of the most fatal types of cancer that is characterized by a limited survival rate: glioblastoma. Using a combination of in vitro and in vivo models of glioblastoma, we showed that the venom effectively killed cancer cells through an unconventional cell death mechanism. These results were observed from cultures of U87 glioblastoma cell lines and transplanted U87 cells in mouse brains, mimicking the tumor in a living organism and allowing for interaction with a microenvironment.

At the cellular level, our findings align with those of previous studies that investigated the effects of *A. mellifera syriaca* venom on various cancer cell lines such as MCF-7, Hela, and HCT-116 cells [38,40,41], indicating its potential as a treatment for various types of cancer. The major component of bee venom, melittin, has the capacity to regulate certain key genes associated with glioma prognosis in U87 cells, which could be responsible for the beneficial cytotoxicity observed [43,44].

At the molecular level, our in vitro results suggested that the venom impairs membrane integrity, a well-known mechanism of melittin [45], the major component of *A. mellifera syriaca* venom [40]. This leads to cellular stress, promoting necrosis; this process can be detected by measuring PARP cleavage into a 55kDa fragment [42]. Interestingly, the in vivo results also showed an increase in caspase-3 levels in the tumor site, suggesting the occurrence of venom-induced apoptosis. These results are not surprising since bee venoms, and melittin in particular, have been shown to induce apoptosis in different cancer cells [44,46]. Necrosis itself does not directly lead to the activation of caspase-3. However, the cellular stress and damage that lead to necrosis can sometimes trigger signals that activate apoptotic pathways, including the activation of caspases [47]. Another possible mechanism is that cells undergoing necrosis release factors that induce nearby cells to undergo apoptosis, potentially involving caspase-3. Alternatively, since the venom contains a variety of molecules, we cannot exclude the possibility of necrotic and apoptotic compounds within the venom.

The capacity of the venom to activate apoptotic pathways in cancer cells, thereby impairing cell death, is of great interest. These results are in line with previous findings reporting that bee venom triggers autophagy-induced apoptosis in human lung cancer cells via the mTOR signaling pathway [48]. The observed increase in apoptotic U87 cells following *A. mellifera syriaca* bee venom treatment can be linked to the molecular mechanisms underlying the action of bee venom components. For example, it has been demonstrated that bee venom induces the interaction between phosphorylated histone variant H2AX and the intracellular site of β-actin in liver and breast cancer cells, indicating its involvement in modulating intracellular signaling pathways related to apoptosis [49]. Moreover, this study highlighted the enrichment of cell membrane signaling pathways in U87 cells, suggesting that bee venom may exert its effects through interactions with cell membrane components and associated signaling pathways [49]. In addition to the direct effects on cancer cells, bee venom has also been shown to modulate cellular pathways and processes that are relevant to cancer progression. For instance, it was reported that bee venom detoxification resulted in increased anti-inflammatory activity and decreased cytotoxicity, indicating its potential to influence the tumor microenvironment and immune responses [50]. Furthermore, the study demonstrated the protective effect of bee venom against ethanol-induced hepatic injury through the regulation of the mitochondria-related apoptotic pathway, highlighting the multifaceted impact of bee venom on cellular survival and apoptosis [51].

The diverse components in bee venom, including melittin, phospholipase A2, apamin, mast cell degranulating peptide, and enzymes, have been shown to exert multifaceted effects on cellular processes and signaling pathways [52]. For instance, melittin has been reported to induce apoptosis in cancer cells through both intrinsic and extrinsic pathways, involving the mitochondria-mediated death signaling cascade and the activation of death receptors [52]. Additionally, bee venom phospholipase A2 has been demonstrated to synergistically generate tumor lysates, enhancing the maturation of immunostimulatory human monocyte-derived dendritic cells, thereby modulating immune responses and potentially contributing to apoptosis induction [53]. Moreover, components of bee venom have been implicated in the regulation of cellular pathways associated with apoptosis. Additionally, bee venom has been reported to trigger autophagy-induced apoptosis in human lung cancer cells via the mTOR signaling pathway, highlighting its ability to modulate key cellular pathways involved in apoptosis regulation [54]. It should also be noted that melittin, the most promising molecule present in the venom of *A. mellifera syriaca*, has already revealed its pharmaceutical potential in the treatment of cancer. This molecule is distinguished by its dual mechanism of action: cell lysis on the one hand, and interaction with cell signaling pathways on the other. A previous study demonstrated melittin’s ability to induce tumor cell apoptosis and act synergistically with first-line therapies, positioning this molecule as a good drug candidate for clinical trials [55]. Melittin was previously found to be highly abundant in *A. mellifera syriaca* venom [40] and could also be the molecule responsible for the anti-cancer effect of the raw venom on glioblastoma observed in this study.

## 4. Conclusions

In this study, we described the anti-cancer potential of *A. mellifera syriaca* venom using in vitro and in vivo glioblastoma models. Our results showed strong toxic effects of the venom on glioblastoma tumors that align with previous findings highlighting the effects of bee venom on cancer cells, and they provide valuable insights into the potential mechanisms and cellular pathways involved in the observed cytotoxicity. This includes the expression of necrosis and apoptosis molecular markers following bee venom treatment. Our results warrant further investigation into the specific molecular targets and signaling pathways affected by bee venom in glioma cells. Such investigations could contribute to the development of novel therapeutic strategies for glioblastoma and other cancer types.

## 5. Materials and Methods

### 5.1. Materials

Human U87 glioblastoma cells were purchased from ATCC (Gaithersburg, MD, USA). DMEM and a penicillin–streptomycin stock were purchased from Invitrogen (Paris, France). DMSO was purchased from Merck (Darmstadt, Germany). MTT, Triton X-100, and an LDH-based cytotoxicity kit were purchased from Sigma-Aldrich (Saint-Quentin-Fallavier, France). Ninety-six-well plates were purchased from Cambridge Technology (Labège, France). Lipofectamine 3000 reagent was purchased form Thermo Fisher Scientific (Waltham, MA, USA). C57BL/6 mice were purchased from Charles River Laboratories. Immunohistochemistry primary antibodies for EGFP (ab290, 1/500 dilution) and caspase-3 (ab208161, 1/500 dilution) were purchased from Abcam.

### 5.2. Venom Collection

Healthy hives of local *A. mellifera syriaca* bee strains were selected for our study. The apiary was located in the central region of Lebanon. The forage came mainly from wild plantations in the region, and the flowers were in full bloom. Venom was collected from healthy, clean colonies of local strains of *A. mellifera syriaca*. Sufficient pollen was available in the area and in the hives (two sets of pollen were available for each colony). Collection was carried out locally using the standard electroshock method [40] at the top of the hive. When the cords were electrified, a light shock was applied to the bees; they covered the surface of the wired glass plate and stung the surface of the glass plate in response to the electrical stimulation. The venom secreted by the bees quickly dried when exposed to air. The dried venom was scraped off with a sharp scalpel and transferred to the laboratory, where it was stored at −20 °C until further analysis. Extraction was performed for 15–20 min on each colony and repeated twice every two weeks.

### 5.3. Cell Culture and Spheroid Formation

U87 glioblastoma cells were cultured in a controlled environment, and were maintained in Dulbecco’s Modified Eagle Medium (DMEM) supplemented with 10% fetal bovine serum (FBS) and 1% penicillin–streptomycin. The cells were routinely subcultured to ensure optimal growth conditions. For injection into mice, U87 cells were transfected using the Lipofectamine 3000 reagent, following the manufacturer’s protocol, to introduce EGFP vectors into the cells. The transfection efficiency was monitored through fluorescence microscopy. To emulate the three-dimensional architecture characteristic of tumors, U87 human glioblastoma cell spheroids were generated. Briefly, the cells were trypsinized, counted, and then seeded into ultra-low attachment plates to encourage spheroid formation. The spheroids, expressing EGFP post-transfection, were cultured in a serum-free medium to maintain their tumor-like properties. The concentration of spheroids for injection was 20,000 cells/mL.

### 5.4. Glioblastoma Cell Proliferation

Cell growth was assessed using the 3-(4,5-dimethylthiazol-2-yl)-2,5-diphenyltetrazolium bromide (MTT) assay, which is based on the reduction of MTT to blue formazan by mitochondrial dehydrogenase in viable cells. U87 cells were seeded into a 96-well plate at a density of 5000 cells/well and incubated overnight at 37 °C. Subsequently, the medium was replaced with a fresh medium containing varying levels of crude bee venom dissolved in PBS or an equivalent amount of venom-free PBS for control cells. After 72 h of treatment, the medium was replaced with 50 μL of 0.5 mg/mL MTT and the cells were incubated at 37 °C for 3 h. After removing the supernatant, the formazan crystals were dissolved using 100 μL of DMSO and the absorbance was read at 600 nm using a spectrophotometer. Viability was calculated by dividing the mean absorbance of the treated cells by that of untreated cells, representing the data as a percentage. Three experiments were conducted (*n* = 3).

### 5.5. Annexin V/Propidium Iodide Staining

U87 cells were plated into 6-well plates at a density of 2.5 × 10^5^ cells per well and incubated at 37 °C overnight. The cells were then treated with 12.5 or 25 µg/mL of bee venom for either 6 h or 24 h. Cells treated with an equivalent amount of venom-free PBS or 50 µM cisplatin were used as negative and positive controls, respectively. Cells were harvested using the classical trypsin protocol, rinsed with PBS, and then resuspended in Annexin V buffer. The cells were labeled with annexin V and propidium iodide for 15 min in the dark following the provider’s recommendations (ab14085), and then processed by flow cytometry (MACSQuant^®^ Analyzer 10 Flow Cytometer, Miltenyi Biotec B.V. & Co. KG, Bergisch Gladbach, Germany).

### 5.6. Cytotoxicity

The impact of the bee venom on cellular integrity in vitro was evaluated through the lactate dehydrogenase (LDH) assay [56]. U87 cells were seeded at a density of 25,000 cells per well into 96-well plates and incubated for 24 h at 37 °C. Subsequently, the culture medium was replaced with fresh media containing varying concentrations of crude bee venom dissolved in PBS. Equivalent amounts of venom-free PBS were used for the control cells. After 24 h of incubation, the supernatants were collected, clarified by centrifugation for 5 min at 600× *g*, and tested using an LDH-based cytotoxicity kit. The total LDH concentration was assessed by adding 0.1% Triton X-100 to untreated cells.

### 5.7. C57BL/6 Mouse Handling, Tumor Injection, and Treatment Procedures

C57BL/6 mice were maintained under controlled conditions, with a standard diet and a 12 h day/night cycle at 25 °C. The animal care adhered to European Community guidelines (2010/63/UE), and was approved by the ethics committee of the Lebanese University. For in vivo experiments, the mice underwent stereotaxic injections of U87 cell spheroids expressing EGFP at a concentration of 20,000 cells/mL. The precise injection coordinates (anteroposterior: −0.1 mm; mediolateral: 1.5 mm; dorsoventral: 0.5 mm) were meticulously calculated to minimize tissue damage. Following a 3-day recovery period, the experimental groups were treated with either a saline solution or 25 µg/mL of bee venom, a concentration selected based on prior in vitro studies.

### 5.8. Immunohistochemistry

For the immunohistochemistry analysis, the mice were euthanized using either a gradual increase in CO_2_ concentration or cervical dislocation. Intracardial perfusion with 1X PBS, followed by fixation with 4% paraformaldehyde (PFA), was carried out to preserve brain tissue integrity for the subsequent brain extraction. The extracted brain tissues were then sectioned into 40 µm slices using a Leica vibratome (Danaher Corporation, Washington, DC, USA). Immunohistochemical staining was performed as described in [57] using primary antibodies against EGFP (Abcam, ab290, 1/500) and caspase-3 (Abcam, ab208161, 1/500).

### 5.9. Hematoxylin and Eosin Staining

A second series of sections were first rehydrated by passing them through a graded alcohol series (100%, 95%, and 70% for 2 min each) and then immersed in Harris Hematoxylin solution for 5 min. Next, the samples were rinsed under running tap water for 5 min to remove any excess stain. For differential staining, the sections were briefly dipped in 0.3% acid alcohol (1% HCl in 70% ethanol) and rinsed again. The hematoxylin staining was intensified by dipping the sections in Scott’s tap water (for 2 min), followed by a final rinse in tap water. The sections were then counterstained with eosin Y for 2 min and briefly rinsed in distilled water to remove any excess stain. The sections were then dehydrated through a series of graded alcohol concentrations and cleared in xylene to remove any remaining alcohol and make the tissue transparent. The stained sections were mounted on glass slides using a synthetic resin mounting medium and allowed to dry completely. Microscopic examination of the stained sections was then performed, and images were captured to compare the cellular mass of U87 cells treated with saline or bee venom three days post-treatment.

### 5.10. Confocal Microscopy Analysis

The analysis of the EGFP expression patterns and apoptotic cell distribution within the brain tissues was conducted using a Zeiss 500 confocal microscope (Oberkochen, Baden-Württemberg, Germany). Laser excitation wavelengths, emission filters, and pinhole settings were optimized for each fluorochrome, ensuring high-resolution imaging and minimizing bleed-through. The quantitative analysis involved the random selection of three 20X fields per section. Data were collected from five animals per experimental group to achieve statistical robustness.

### 5.11. Protein Extraction and Western Blot

U87 cells were trypsinized, counted, and seeded at a density of 175,000 cells per well into a 6-well plate and cultured overnight. The next day, the culture media was replaced with fresh media containing 50 nM cisplatin or bee venom (12.5 µg/mL or 25 µg/mL). Twenty-four hours after treatment, the cells were washed twice with TBS and lysed in RIPA buffer (50 mM Tris, 0.1% SDS, 1 mM EDTA, and 1% Triton, supplemented with protease and phosphatase inhibitors) for 30 min. The lysates were then centrifuged at 14,000× *g* for 15 min. The protein content in the supernatant was quantified using the Biorad Bradford method with BSA as the standard. The lysates were diluted using NuPAGE LDS sample buffer (Merck, Rahway, NJ, USA) and stored at −20 °C until further use.

For Western blotting, 12.5 µg protein samples were separated on a 10% SDS-PAGE gel and subsequently transferred onto a nitrocellulose membrane. The membrane was then blocked with TBS containing 5% milk and 0.15% Tween-20 for 1 h at room temperature. Following the blocking step, the membranes were incubated overnight at 4°C with primary antibodies: anti-PARP (cell signaling, 9542S) at 1/1000 and anti-GAPDH (G8795) at 1/5000. After three washes with TBS containing 0.15% Tween-20, the membranes were incubated with horseradish peroxidase-conjugated anti-rabbit (cell signaling, 7074S) or anti-mouse (Cell signaling, 7076S) secondary antibodies for 1 h. The membranes were then washed three times with TBS containing 0.15% Tween-20 and once with TBS before incubation with the Immobilon Western chemiluminescent HRP substrate. Chemiluminescence was detected and captured using a chemiluminescence imaging system.

### 5.12. Statistical Analyses

The data were analyzed using GraphPad Prism V8 (GraphPad Software, San Diego, CA, USA) and are represented as the mean ± SD of at least 3 independent experiments performed in triplicate for each condition. One-way ANOVA was performed. Differences were considered significant at *p* < 0.05 (*), *p* < 0.01 (**), *p* < 0.001 (***), and *p* < 0.0001 (****). 

## Figures and Tables

**Table 1:** Summary of *A. mellifera syriaca* venom's main components and properties that were identified in our previous study [40] along with their biological functions (aa: amino acid; Da: Dalton; “+”: present and “+++”: highly present/abundant). Of note, this list is not exhaustive as different technical and environmental factors can affect the identified compounds.

Molecule	Composition, Molecular Weight	Abundance	Function
Apamin	18 a.a., 2027 Da	+	Neurotoxin
Melittin	26 a.a., 2846.4 Da	+++	Main component and the major pain-producing substance of honeybee venom
Phospholipase A2	128 a.a., 18,964 Da	+++	Enzyme responsible for phospholipid hydrolysis
MCD-peptide	22 a.a., 2599.8 Da	+	Potent and selective blocker of KV1.1 and KV1.2 channels
Hyaluronidase	349 a.a., 53,875.6 Da	+	Enzyme that temporarily and reversibly depolymerizes hyaluronic acid

## Data Availability

Data are contained within the article.
